# Multivariate Analysis as a Method for Evaluating the Conceptual Perceptions of Korean Medicine Students regarding Phlegm Pattern

**DOI:** 10.1155/2013/761497

**Published:** 2013-08-26

**Authors:** Hyungsuk Kim, Hyunho Kim, Young-Jae Park, Young-Bae Park

**Affiliations:** ^1^College of Korean Medicine, Kyung Hee University, Seoul 130-702, Republic of Korea; ^2^Department of Biofunctional Medicine & Diagnostics, College of Korean Medicine, Kyung Hee University, Seoul 130-702, Republic of Korea

## Abstract

Individuals may perceive the concepts in Korean medicine pattern classification differently because it is performed according to the integration of a variety of information. Therefore, analysis about individual perspective is very important for examining the cross-sectional perspective state of Korean medicine concepts and developing both the clinical guideline including diagnosis and the curriculum of Korean medicine colleges. Moreover, because this conceptual difference is thought to begin with college education, it is worthwhile to observe students' viewpoints. So, we suggested multivariate analysis to explore the dimensional structure of Korean medicine students' conceptual perceptions regarding phlegm pattern. We surveyed 326 students divided into 5 groups based on their year of study. Data were analyzed using multidimensional scaling and factor analysis. Within-group difference was the smallest for third-year students, who have received Korean medicine education in full for the first time. With the exception of first-year students, the conceptual map revealed that each group's mean perceptions of phlegm pattern were distributed in almost linear fashion. To determine the effect of education, we investigated the preference rankings and scores of each symptom. We also extracted factors to identify latent variables and to compare the between-group conceptual characteristics regarding phlegm pattern.

## 1. Introduction

In western medicine, the physiology, pathology, and concepts of disease are described using quantitative and objective descriptive terms of molecular biology, histophysiology, and natural science, whereas those of Korean medicine are depicted in relatively abstract terms based on old Chinese ideographic characters. Pattern classification, a unique diagnostic method of Korean medicine, is a significant holism-based tool used to extract and integrate the sign and symptom information of patients for diagnosis. However, pattern classification terms are in fact abstractive because since *Huangdi's Internal Classic*, which is acknowledged as having been written almost 2000 years ago, various terms of Korean medicine have been created, disappeared, or slightly changed by many medical groups. Therefore, practitioners could have different images about the same pattern concepts, and it is a challenging issue in professional education or in academic discussion in Korean medicine.

Many studies have been conducted to overcome these concept-related communication problems. For example, the World Health Organization project to derive consensus regarding the standardization of traditional medicine terminology [1] and the development of ontology by the Korea Institute of Oriental Medicine for medicinal materials based on Korean medicine [2] are studies to render abstract concepts more objective. In addition, there have been efforts towards objectification of tongue diagnosis [3] and pulse diagnosis [4] based on the use of diagnostic instruments. Questionnaires have also been developed to diagnose several patterns such as Yin-deficiency [5], phlegm pattern [6], food accumulation, 7 emotions, overexertion and fatigue, static blood, and subhealth [7] using modern statistical methodology such as factor analysis, principal component analysis, or structural equation. Data from diagnostic devices or questionnaires, however, cannot present the corresponding pathologic concept perfectly. Thus, a practitioner's clinical decision is still the most important, and the decision of pattern classification generally comes from the conceptual perception formed by the college education [8]. For these reasons, it is necessary to measure the conceptual perception of individuals, especially students. To evaluate it, a new technique would be helpful to describe and compare these invisible conceptual perceptions.

Multidimensional scaling (MDS) is a statistical and visualization method used to project multidimensional variable objects into lower dimensions to analyze the association between the objects. It can also model objects and evaluators together in the same conceptual space, enabling analysis of the between- or within-evaluator difference of perception. There are two approaches in MDS. One is the similarity-analyzing method based on a similarity matrix, and the other is a method for evaluating the objects and ideal point (or ideal vector) simultaneously based on the preference matrix [9–11]. MDS has mainly been applied to the field of marketing to observe customer awareness and to conduct positioning of new products and is used in education as well because it visualizes the abstract perception of learners. Several studies on medical students have used this method to study students' perception of physical symptoms [12], students' personal and professional development [13], students' views on empathy in medical education [14], and to visualize the dimensional structure of medical students' perceptions of diseases [15]. There have also been MDS studies on traditional medicine: observation of the association between the cold and heat pattern of traditional tongue diagnosis and the tongue coating microbiome [16], and study of the similarity and dissimilarity of pattern analysis by physicians regarding patients' tongue diagnosis information [17]. Meanwhile, factor analysis explains the structure of items, finding latent variables that affect multivariable data. It is usually used when developing questionnaires, such as verifying the construct validity of a questionnaire and grouping variables according to latent factors. Some studies have used factor analysis in traditional medicine to study the attitudes and skills of Hong Kong Chinese medicine practitioners towards computerization in practice [18], the attitudes of Hong Kong Chinese medicine practitioners to traditional Chinese medicine and western medicine [19], the development of a stagnation questionnaire [20], factor analysis of the symptoms of unstable angina [21], chronic low back pain [22], dysfunctional uterine bleeding [23], and efficiency study of the cold and heat pattern in the symptoms of rheumatoid arthritis [24]. Nevertheless, there have been no studies to evaluate and compare their perceptions of concepts in Korean medicine.

Therefore, we conducted a study to evaluate and analyze the individual concepts of phlegm pattern numerically and visually and applied these methods to the Korean medicine students. Using an internationally published phlegm pattern questionnaire and the above mentioned methods, we could compare the perceptive structure of the students' concepts regarding phlegm pattern and discuss the differences in the educational aspects.

## 2. Materials and Methods

### 2.1. Subjects

We tried to perform a complete enumeration survey of the first- to fifth-year students of the College of Korean Medicine, Kyung Hee University, Seoul, Korea. The College of Korean Medicine teaches a 6-year course: students are taught general education and introduction to Korean medicine during the first and second years, while full Korean medicine education begins in the third year. However, sixth year students does not take any classes, they only do practice in hospital. Thus, students from first to fifth year were included in this study. Data were acquired during December 2012, which is the period in which each year of study finishes. We provided proper gifts as rewards to encourage honest and sincere responses. The students were requested to judge the importance level of each symptom to diagnose phlegm pattern according to 5-point Likert scale: 1, “very insignificant”; 2, “insignificant”; 3, “moderate”; 4, “significant”; 5, “very significant.” Three hundred and sixty four students answered the survey; the data of 38 students were excluded due to the missing values. Finally, 326 students' records were analyzed out of 542 students in the whole school excluding sixth-year students (60.1%). The characteristics for participants are listed in Table 1.

### 2.2. Phlegm Pattern Questionnaire

The phlegm pattern questionnaire was developed in 2011 to evaluate a patient's phlegm pattern score and consists of 25 items: 7 neuropsychologic, 4 respiratory, 3 fatigue-related, 5 gastrointestinal, 4 dermatological, and 2 pain-related symptoms. The items of the phlegm pattern questionnaire are presented in the Supplementary Material (See Supplementary Material available online at http://dx.doi.org/10.1155/2013/761497). Cronbach's alpha was 0.919 and the item-total correlations of all items were >0.3 [6]. Structural validity was examined by factor analysis with varimax rotation, and the first 6 factors explained 58.9% of the total variance. According to the receiver operating characteristic curve, the cut-off point was calculated to be 5. At that level, the sensitivity was 83.78% and the specificity was 83.33%.

### 2.3. Statistical Parameters and Analysis

#### 2.3.1. Distance Matrix and Parameters

To perform MDS analysis, the distance matrix between the subjects should be calculated to present them in perceptual space. Some types of distance are generally calculated according to the properties of the data, and thus we used Euclidean distance to obtain the distance matrix in this study

(1) $$\begin{array}{c} {\text{PD}}_{ij}=\sqrt{\overset{25}{\underset{n=1}{\sum }}{\left(A_{in}-A_{jn}\right)}^{2}}, \end{array}$$

PD_*ij*_ is the Euclidean pair distance between 2 arbitrary objects, *i* and *j*. *A*_*in*_ is the response score of the *n*th questionnaire item by object *i*. Based on these PD_*ij*_ values, we were able to calculate the mean value of all pair distances using the following equation, where *N* is the number of students of each year. We applied this equation to all 5 groups, which were defined by the year of study

(2) $$\begin{array}{c} \overset{-}{\text{PD}}=\frac{\sum_{i=1}^{N}\sum_{j=1}^{N}{\text{PD}}_{ij}}{N\left(N-1\right)/2}. \end{array}$$

Separately, absolute distance (AD), a student's perceptive distance from the average point of the corresponding year of study, was calculated using the following equation. ${\overset{-}{A}}_{n}$ indicates the mean of the *n*th questionnaire item and *i* is the index of a student. The mean and standard deviation of the AD were assessed. These calculations were performed for each year of study

(3) $$\begin{array}{c} {\text{AD}}_{i}=\sqrt{\overset{25}{\underset{n=1}{\sum }}{\left(A_{in}-{\overset{-}{A}}_{n}\right)}^{2}}. \end{array}$$

#### 2.3.2. MDS and Factor Analysis

MDS is a statistical and visualization method used to map a set of multivariable data to a lower dimensional space for the convenience of intuitive insight or understanding of the data. A 2- or 3-dimensional model is typical because of the limitation of man's spatial perception. MDS is divided into two approaches: one to visualize the similarity of the data, and the other to calculate the ideal point or ideal vector additionally using preference data. As we intended to present within- and between-group similarity, we used the ALSCAL algorithm, a dimension-lowering algorithm with conserving between-object distances. Factor analysis is a modern statistical method that allows the determination of latent variables from directly measurable variables. The Kaiser criterion was used and the factors with eigenvalues ≥ 1.00 were retained. After principal component extraction, varimax rotation was performed. All statistical calculations were performed using SPSS Statistics 19 (SPSS Inc., Chicago, IL, USA) and Excel 2007 (Microsoft Office Excel 2007; Microsoft, Redmond, WA, USA).

## 3. Results and Discussion

### 3.1. Within-Group and Between-Group Distribution of Conceptual Perception of Phlegm Pattern

As shown in Table 2, the mean within-group pair distance values of conceptual perception of phlegm pattern in the Euclidean system were 5.98–6.79. Analysis of variance (ANOVA) revealed that statistically significant differences exist among the 5 groups (*P* < 0.001). Dunnett's T3 test, a post hoc analysis of unequal-variance assumed data, was used to analyze the homogeneous subgroups indicated in Table 2. First- and second-year students belonged to one subset, and fourth- and fifth-year students belonged to another subset. Third-year students had a statistically narrow distribution in comparison with the other 4 groups. This means that some consensus regarding phlegm pattern was formed among third-year students possibly due to the beginning of full Korean medicine education. The within-group conceptual gap increased again from the fourth year. This may be owing to the influence of the various Korean medicine classics or various pattern classification systems, because Korean medicine theories emphasize diagnostic points in a slightly different manner. However, the distances were statistically shorter than that of the first- or second-year students.

$\overset{-}{\text{AD}}$ value, the mean distance from the average point of each year of study, exhibited a similar tendency. As the statistical significance was not revealed (*P* = 0.093), this should be considered from the exploratory viewpoint.

**Figure 1:**
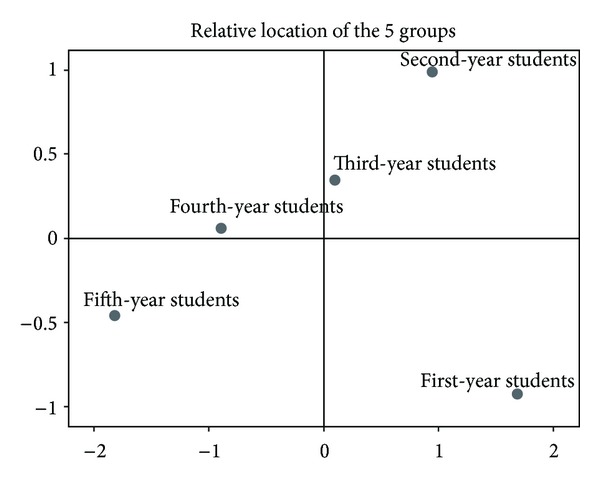
Between-group distribution of conceptual perception of phlegm pattern.

Figure 1 illustrates a 2-dimensional model of the mean points of phlegm pattern conceptual perception of the 5 groups according to the Euclidean distance matrix by ALSCAL algorithm. The stress value of this model was 0.00545 and the *R* ^2^ was 0.99978. This indicates that the goodness of fit of this model was very high. The important physical value is the relative distance between 2 arbitrary points; therefore, the 2 axes are physically meaningless. As shown in the figure, the first- and second-year students are furthest from the fifth-year students, and the distance between them is very long. The second- to fifth-year students are aligned in order in a relatively straight line. This figure expresses that the conceptual gap between the fourth- and the fifth-year students is small compared with that between the third- and fifth-year students or between the second- and fifth-year students. This also demonstrates that the conceptual perception of phlegm pattern is not formed correctly in the first and second year of study, but as the year of study increases, the average perceptive distance of each group from the fifth-year students' perception decreases. In other words, full Korean medicine education is believed to result in the average conceptual perception of each year of study resembling that of fifth-year students.

### 3.2. Symptom Preference for Phlegm Pattern Diagnosis according to Year of Study

**Figure 2:**
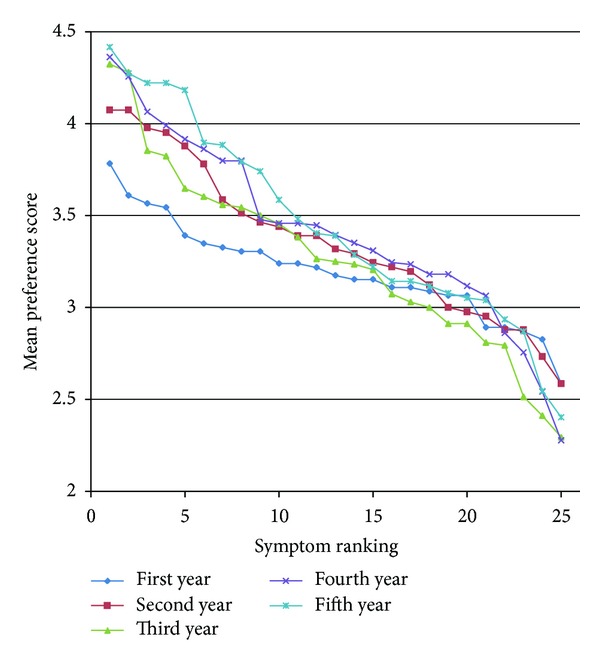
Symptom preference score for phlegm pattern diagnosis.

Figure 2 depicts the average score of each questionnaire item according to year of study. A high score meant “this symptom is very important for diagnosing a patient as phlegm pattern.” According to Figure 2, the difference of symptom importance becomes clearer as the year of study increases. The score difference between the most and least important symptoms was 2.01 (fifth-year students) and 1.20 (first-year students). The difference between the average scores of 5 most and 5 least important symptoms was 1.50 (fifth-year students) and 0.77 (first-year students). The difference had a tendency to increase with the year of study. We may consider that the weight value of symptoms differs largely based on the year of study; that is, some certain symptoms are believed to be more important than others as the year of study increases.

Table 3 lists the differences in preference of symptoms according to year of study for phlegm pattern diagnosis. ANOVA was used to test the statistical significance. Twenty-five symptoms were tested as independent events; therefore, we adjusted the alpha level as 0.05/25 by Bonferroni correction to compensate for accumulated alpha error. With this, we could guarantee that the total alpha error <0.05. At this level, a statistically significant difference was observed for the symptoms: “unclearness in the head,” “feeling of foreign body in the throat,” “sputum,” “sickness,” “indigestion,” “feeling of abdominal fullness,” “dizziness,” and “lumps.” Then, we tested these symptoms again using Scheffe's or Dunnett's T3 post hoc analysis based on the variance homogeneity of the data. Homogeneous subsets were grouped by these statistical methods. Symptoms with *P* < 0.05 were also indicated for exploratory consideration even though they were unable to meet the adjusted *P* value condition of under 0.002.

“Feeling of foreign body in the throat” and “sputum” ranked highly in the average score and rank for all years of study. Moreover, there was a tendency for these items to score higher as the year of study increased. From this, we believe that all students had the impression that phlegm pattern is highly correlated with symptoms of the throat. “Phlegm” directly indicates a secretion, that is, “sputum”. This might have been the reason for the high preference for “sputum” and “feeling of foreign body in the throat” in the diagnosis of phlegm pattern. “Fatigue” scores were similar for all years of study, but its ranking fell from the second year onwards. This indicates that the students similarly preferred this symptom, but came to believe that other symptoms were more important in evaluating phlegm pattern as the year of study increased. The scores and rankings for “sickness” and “dizziness” showed a tendency to increase, especially rapidly from the second to third year. Based on this, we may believe that third-year students have been taught that these symptoms are very important and featured in evaluating phlegm pattern.

All years of study felt that “Feeling heavy in the limbs,” “startled by faint noise,” “palpitation,” and “itching” were not particularly important in phlegm pattern. Only the fifth-year students registered a high preference for “dark circle under the eye,” whilst its score and ranking by the first- to fourth-year students were almost identically low. 

There was a unique phase for “lumps,” where it was scored and ranked very highly by the second-year students. It is possible there had been a lecture or event that had classified “lumps” as being very important and characterized in phlegm pattern. The third- to fifth-year students registered low scores and ranks for “lumps”; therefore, we believe that only the second-year students had this experience, or that the full Korean medicine education beginning in the third year corrected this overestimated importance. 

Symptoms that scored a mean value <3.0 were thought to have “no or less significance” for evaluating or diagnosing phlegm pattern. Seven symptoms were scored <3.0 at least once by the third-, fourth-, or fifth-year students, and 3 symptoms were scored <3.0 in all of these 3 groups. We excluded the first- and second-year students in this discussion because they had not been taught full Korean medicine yet. There are 2 possible reasons for the low mean values. First, it is possible that education regarding these symptoms is not conducted enough, although all 25 symptoms are almost equally significant for diagnosing phlegm pattern according to Park et al.'s study [6]. Second, it is also possible that, in practice, these symptoms have less significance than other symptoms for diagnosing phlegm pattern; thus, the teaching process places less emphasis on them. Further studies should be conducted to explain this phenomenon properly. 

### 3.3. Factor Analysis

Tables 4, 5, 6, 7, and 8 present the factor loadings and results of factor analysis regarding the response of the phlegm pattern questionnaire according to the year of study. The factors were constructed using items with a factor loading ≥0.4 for the exploratory factor analysis.

Table 9 lists the extracted factors for the between-group comparison. For the convenience of comparison, the factors are listed with similar factors in a row regardless of their variance explained. The rightmost column contains the factors extracted from the health information of existing patients by Park et al. [6]. 

With the exception of the fourth-year students, “sputum” and “cough” were grouped together by all years of study. Similarly, with the exception of the fifth-year students, “feeling of foreign body in the throat” and “sputum” were extracted into the same factor. Hence, the students apparently consider “sputum,” “cough,” and “feeling of foreign body in the throat” as one factor, that is, a respiratory system problem. For all years of study, “sickness,” “indigestion,” and “poor appetite” were grouped together. We believe that this factor is digestive system problem. For all years of study, “fatigue” and “feeling heavy in the limbs” were grouped together. As these 2 symptoms are the typical symptoms of qi-deficiency pattern, one of the Korean medicine pattern classifications, they exhibit a high correlation. The fourth- and fifth-year students grouped “poor appetite” with “fatigue,” “feeling heavy in the limbs,” and “shortness of breath,” and these symptoms are also related to qi-deficiency pattern. In other words, the fourth- and fifth-year students consider “poor appetite” a symptom of qi-deficiency pattern unlike the first- to third-year students. This may be the effect of the full Korean medicine education. Only the first-year students separated “feeling of abdominal fullness” from “sickness” and “indigestion.” Perhaps these students have not yet formed the concept of relating “feeling of abdominal fullness” with the latter 2 symptoms as a digestive system problem. All years of study perceived “headache” and “unclearness in the head” as the same factor. “Palpitation” and “startled by faint noise” also fell within the same factor for all years of study. These 2 symptoms are known as symptoms of the mind in Korean medicine and are treated with similar prescriptions. Thus, this may have affected the students' conceptual perception.

Generally, the factor analysis results of the students' conceptual perception are similar to that of Park et al.'s [6]. Accordingly, we may believe the students' concept of phlegm pattern has a similar dimensional structure containing the manifesting pattern of symptoms in patients. 

### 3.4. Limitation and Further Study

There are some limitations in this study. First, it could not demonstrate the time effect of education directly, because it was a cross-sectional study focusing on a certain point of time, and not a time-series study performed on the same group students from their entry into college to advancing higher years of study. Second, although the College of Korean Medicine of Kyung Hee University is the biggest Korean medicine college in Korea, it does not represent the dimensional structure of conceptual perception of all Korean medicine students in Korea, as each college possesses a certain amount of distinct educational characteristics and curricula. Further studies of a long-term, time-series study and comparison with students from other colleges should be performed in the future. Then, how the full Korean medicine education can affect the students' perceptions over time can be identified more precisely.

## 4. Conclusion

In this study, we attempted to observe the perceptive characteristics of Korean medicine students' concepts of phlegm pattern according to the year of study mainly by MDS and factor analysis. We found that third-year-student group had the narrowest within-group distribution of perceptions regarding phlegm pattern. Moreover, we were able to observe the difference in diagnostic preference regarding symptoms between years of study. We could witness the apparent effect of education from this study. Finally, by factor analysis, we found that the extracted conceptual factors have similar tendencies with a previously conducted clinical trial study. We expect that this study lends critical meaning to the study of the pattern classification system of Korean medicine and to the study of the structure of conceptual perception in Korean medicine students.

## Supplementary Material

This phlegm pattern questionnaire was developed in 2011 to evaluate a patient's phlegm pattern score and consists of 25 items.
Researchers used Delphi method, factor analysis and ROC curves to develop and validate the questionnaire.

## Figures and Tables

**Table 1:** Characteristics for participants.

	First year	Second year	Third year	Fourth year	Fifth year
Total students	107	113	100	123	99
Refusal	57	65	24	23	15
Participants	50	48	76	100	84
Missing value records	4	7	8	6	7
Finally included records	46	41	68	94	77
Included records					
Age, years (M ± SD)	21.1 ± 1.39	23.4 ± 4.76	23.0 ± 1.97	24.8 ± 3.48	25.4 ± 4.08
Male	25	25	38	63	49
Female	21	16	30	31	28
Response rate	43.0%	36.3%	68%	76.4%	77.8%

M: mean.

SD: standard deviation.

**Table 2:** Within-group distribution of conceptual perception of phlegm pattern.

Year of study	First year	Second year	Third year	Fourth year	Fifth year	*P* value
Mean PD_*ij*_	6.79^a^	6.65^a^	5.98^b^	6.43^c^	6.36^c^	<0.001
SD of PD_*ij*_	1.57	2.34	1.18	1.43	1.26	
Mean AD_*i*_	4.72	4.62	4.18	4.49	4.44	0.093
SD of AD_*i*_	1.24	1.19	0.92	1.12	1.00	

SD: standard deviation.

*i*, *j*: index of a student.

^abc^Indicators of the homogeneous subsets grouped by Dunnett's *T*3 test.

**Table 3:** Symptom preference for phlegm pattern diagnosis.

Symptom	Ranking (score mean)				
	First year	Second year	Third year	Fourth year	Fifth year
Feeling heavy in the chest	1 (3.78)	5 (3.88)	4 (3.82)	3 (4.06)	8 (3.79)
Unclearness in the head^∗1^	2 (3.61)^a^	9 (3.46)^a^	3 (3.85)^ab^	5 (3.91)^ab^	5 (4.18)^b^
Feeling of foreign body in the throat^∗2^	3 (3.57)^a^	1.5 (4.07)^ab^	1 (4.32)^b^	2 (4.26)^b^	2 (4.27)^b^
Sputum^∗2^	4 (3.54)^a^	1.5 (4.07)^ab^	2 (4.28)^b^	1 (4.36)^b^	3.5 (4.22)^b^
Fatigue	5 (3.39)	11.5 (3.39)	13 (3.25)	12 (3.45)	10 (3.58)
Headache	6 (3.35)	10 (3.44)	10 (3.46)	14 (3.35)	14 (3.29)
Yellow face	7 (3.33)	16 (3.22)	18 (3.00)	16 (3.24)	20 (3.05)
Sickness^∗2^	8.5 (3.30)^a^	11.5 (3.39)^a^	5 (3.65)^ab^	4 (3.99)^bc^	3.5 (4.22)^c^
Indigestion^∗1^	8.5 (3.30)^a^	6 (3.78)^b^	9 (3.50)^ab^	7.5 (3.80)^b^	7 (3.88)^b^
Feeling of abdominal fullness^∗1^	10.5 (3.24)^a^	4 (3.95)^b^	8 (3.54)^ab^	7.5 (3.80)^b^	6 (3.90)^b^
Rumbling sound in the abdomen**	10.5 (3.24)	14 (3.29)	19.5 (2.91)	18.5 (3.18)	12 (3.40)
Dizziness^∗2^	12 (3.22)^a^	17 (3.20)^a^	7 (3.56)^ab^	6 (3.86)^b^	1 (4.42)^c^
Cough**	13 (3.17)	8 (3.51)	6 (3.60)	15 (3.31)	16.5 (3.14)
Shortness of breath	14.5 (3.15)	13 (3.32)	11 (3.38)	20 (3.12)	18 (3.12)
Feeling heavy in the limbs	14.5 (3.15)	22.5 (2.88)	21 (2.81)	21 (3.06)	22 (2.94)
Lumps^∗1^	16.5 (3.11)^a^	3 (3.98)^b^	12 (3.26)^a^	10.5 (3.46)^ab^	19 (3.08)^a^
Dark circles under the eyes**	16.5 (3.11)	15 (3.24)	15 (3.21)	17 (3.23)	9 (3.74)
Mucousy stool	18 (3.09)	21 (2.95)	19.5 (2.91)	18.5 (3.18)	21 (3.04)
Poor appetite**	19.5 (3.07)	19 (3.00)	14 (3.24)	9 (3.48)	11 (3.48)
Flank pain**	19.5 (3.07)	7 (3.59)	17 (3.03)	13 (3.39)	16.5 (3.14)
Startled by faint noise**	21.5 (2.89)	25 (2.59)	25 (2.29)	24 (2.54)	24 (2.55)
Joint pain**	21.5 (2.89)	18 (3.12)	16 (3.07)	10.5 (3.46)	13 (3.39)
Palpitation**	23 (2.87)	22.5 (2.88)	24 (2.41)	23 (2.76)	23 (2.87)
Tinnitus	24 (2.83)	20 (2.98)	22 (2.79)	22 (2.86)	15 (3.22)
Itching**	25 (2.59)	24 (2.73)	23 (2.51)	25 (2.28)	25 (2.40)

Symptoms of same ranking scored the median value.

Item that has statistically significant mean difference among groups by analysis of variance.

*P < 0.05/25 by the Bonferroni correction for the multiple comparisons.

**P < 0.05 for the exploratory analysis.

^1^Scheffe's test for the post hoc multiple comparisons of the equal variance assumed data.

^2^Dunnett's *T*3 test for the post hoc multiple comparisons of the unequal variance assumed data.

^abc^Indicators of the homogeneous subsets grouped by Scheffe's test or Dunnett's *T*3 test in a row.

**Table 4:** Factor Loadings for the items of the phlegm pattern questionnaire responded by the first-year students.

Item	Factor								
	1	2	3	4	5	6	7	8	9
Sputum	**0.849**	0.083	0.043	−0.180	0.004	−0.120	−0.255	0.132	0.201
Cough	**0.847**	−0.019	0.196	−0.092	−0.065	0.092	0.083	0.149	0.034
Feeling of foreign body in the throat	**0.799**	0.233	−0.053	0.226	−0.096	−0.149	−0.168	−0.041	0.076
Shortness of breath	**0.626**	0.116	−0.073	0.205	−0.055	0.156	**0.512**	−0.125	−0.104
Indigestion	0.130	**0.840**	0.105	−0.148	0.030	0.091	0.065	0.272	−0.008
Sickness	0.150	**0.788**	−0.071	0.226	−0.086	−0.268	0.035	−0.107	0.053
Lumps	−0.035	**0.605**	−0.031	**0.556**	0.029	0.064	−0.198	0.037	0.244
Poor appetite	−0.029	**0.585**	0.557	−0.013	−0.052	−0.009	0.186	0.179	0.064
Fatigue	0.042	−0.048	**0.830**	0.017	0.005	0.061	0.173	0.088	0.003
Feeling heavy in the limbs	0.030	0.052	**0.793**	0.050	0.194	−0.045	0.243	0.247	−0.151
Yellowish face	0.228	**0.433**	**0.582**	0.137	0.114	−0.124	−0.247	−0.227	−0.185
Tinnitus	0.025	0.012	−0.066	**0.792**	0.081	−0.067	0.299	0.097	−0.133
Dizziness	0.017	−0.038	0.243	**0.703**	0.017	**0.442**	0.085	−0.179	−0.212
Flank pain	−0.385	0.283	0.183	**0.453**	0.367	−0.166	0.110	0.215	0.298
Itching	−0.010	0.016	0.072	−0.121	**0.880**	−0.055	0.020	−0.198	−0.136
Joint pain	−0.183	−0.112	0.104	0.097	**0.811**	0.190	−0.119	−0.001	0.234
Dark circle under the eyes	0.010	0.013	−0.006	0.221	**0.735**	0.152	0.237	0.221	−0.266
Headache	−0.028	−0.008	0.080	0.183	0.051	**0.866**	−0.039	0.133	0.016
Unclearness in the head	0.015	−0.011	−0.083	−0.080	0.100	**0.820**	−0.127	−0.090	−0.005
Mucousy stool	0.148	0.304	0.203	**0.411**	−0.067	**−0.458**	−0.108	0.376	−0.023
Palpitation	−0.028	0.118	0.235	0.262	0.084	−0.132	**0.702**	−0.006	−0.022
Startled by faint noise	−0.235	−0.051	0.297	−0.025	0.022	−0.115	**0.687**	0.272	0.045
Rumbling sound in the abdomen	0.089	0.102	0.192	0.061	−0.014	−0.056	0.074	**0.863**	−0.019
Feeling of abdominal fullness	0.106	**0.529**	0.076	−0.081	−0.061	0.211	0.211	**0.572**	−0.313
Feeling heavy in the chest	0.218	0.067	−0.143	−0.142	−0.106	0.015	0.003	−0.093	**0.878**
**Variance explained (%)**	11.57	11.20	9.61	8.98	8.93	8.80	7.26	7.17	5.40

Symptoms with factor loading ≥0.4 are marked bold.

**Table 5:** Factor loadings for the items of the phlegm pattern questionnaire responded by the second-year students.

Item	Factor						
	1	2	3	4	5	6	7
Unclearness in the head	**0.815**	0.026	0.039	0.036	0.159	−0.013	0.136
Headache	**0.711**	−0.065	−0.027	0.006	0.097	0.394	0.248
Lumps	**0.705**	0.289	−0.087	0.283	0.008	0.098	−0.335
Joint pain	**0.703**	−0.115	−0.101	0.357	0.222	0.067	0.071
Flank pain	**0.641**	0.357	0.268	0.060	−0.144	−0.106	−0.287
Feeling heavy in the chest	**0.563**	0.184	**0.536**	0.034	0.257	−0.331	−0.082
Feeling of abdominal fullness	**0.400**	**0.808**	0.019	0.069	−0.038	0.013	0.123
Indigestion	−0.022	**0.710**	0.373	−0.129	−0.025	−0.046	0.240
Rumbling sound in the abdomen	0.023	**0.617**	−0.190	**0.535**	0.000	0.129	0.013
Sickness	−0.159	**0.584**	0.172	−0.082	0.385	**0.500**	0.162
Poor appetite	−0.228	**0.460**	**0.424**	−0.131	0.217	0.249	0.398
Startled by faint noise	0.081	0.107	**0.855**	0.138	0.063	0.276	−0.006
Palpitation	0.034	0.035	**0.721**	0.276	−0.183	0.310	0.034
Itching	−0.003	−0.110	0.191	**0.808**	−0.021	−0.104	0.085
Dark circles under the eyes	0.238	0.090	−0.013	**0.740**	−0.154	0.199	−0.139
Yellowish face	**0.425**	−0.115	0.240	**0.499**	0.067	0.144	0.323
Mucousy stool	0.297	0.156	0.168	**0.488**	0.157	0.269	0.186
Sputum	0.212	0.060	−0.012	0.036	**0.878**	−0.090	−0.002
Cough	0.004	−0.328	0.047	−0.007	**0.820**	−0.086	0.228
Feeling of foreign body in the throat	0.154	0.241	0.005	−0.081	**0.704**	0.189	0.085
Tinnitus	0.026	0.056	0.275	0.092	−0.034	**0.819**	0.277
Dizziness	0.156	0.052	0.168	0.150	−0.007	**0.810**	−0.076
Feeling heavy in the limbs	−0.011	0.118	0.004	0.015	0.113	0.135	**0.809**
Fatigue	0.230	0.176	−0.049	0.240	0.037	−0.023	**0.733**
Shortness of breath	−0.144	0.091	0.461	−0.174	0.201	0.101	**0.535**
**Variance explained (%)**	14.513	10.606	9.835	9.819	9.819	9.375	9.315

Symptoms with factor loading ≥0.4 are marked bold.

**Table 6:** Factor loadings for the items of the phlegm pattern questionnaire responded by the third-year students.

Item	Factor							
	1	2	3	4	5	6	7	8
Feeling of foreign body in the throat	**0.688**	0.183	−0.285	−0.032	0.160	0.024	0.098	−0.085
Cough	**0.686**	−0.064	−0.081	−0.087	−0.210	−0.049	−0.052	0.338
Sputum	**0.640**	0.043	−0.171	**−0.415**	−0.180	0.110	0.071	0.227
Shortness of breath	**0.639**	0.141	0.104	**0.449**	0.004	−0.132	0.055	0.131
Feeling heavy in the chest	**0.628**	0.000	0.189	0.089	0.201	0.057	0.074	−0.288
Sickness	0.221	**0.796**	0.063	0.094	−0.045	−0.046	−0.192	−0.103
Indigestion	0.028	**0.774**	−0.081	−0.094	0.081	0.114	0.315	−0.127
Feeling of abdominal fullness	0.062	**0.676**	−0.248	−0.078	0.238	−0.017	**0.411**	−0.091
Poor appetite	−0.109	**0.634**	0.306	−0.211	−0.016	0.152	−0.127	0.171
Feeling heavy in the limbs	−0.167	−0.058	**0.835**	0.118	−0.055	0.131	0.113	−0.067
Fatigue	0.000	0.128	**0.738**	0.042	0.200	0.125	−0.075	−0.016
Palpitation	−0.092	−0.003	0.227	**0.727**	0.024	0.062	−0.114	0.180
Startled by faint noise	0.118	−0.221	−0.052	**0.712**	0.186	0.225	−0.077	−0.202
Tinnitus	0.013	−0.059	−0.040	**0.547**	−0.116	**0.542**	0.193	0.032
Yellowish face	−0.036	0.203	−0.154	−0.042	**0.747**	0.038	−0.086	0.012
Joint pain	−0.006	−0.060	0.366	0.083	**0.698**	−0.016	−0.124	0.156
Flank pain	0.144	−0.119	**0.444**	0.156	**0.567**	−0.062	−0.105	0.130
Dark circles under the eyes	−0.315	0.106	0.318	0.250	0.345	0.048	0.266	0.217
Unclearness in the head	0.033	0.011	0.164	0.015	0.046	**0.850**	−0.132	−0.051
Dizziness	−0.231	0.101	0.132	0.355	−0.133	**0.625**	0.055	0.120
Headache	0.227	0.323	0.252	0.119	0.236	**0.484**	−0.142	0.366
Lumps	0.252	0.113	−0.184	−0.329	0.330	**0.434**	0.313	−0.135
Mucousy stool	0.164	−0.097	−0.026	−0.071	−0.187	0.052	**0.794**	0.008
Rumbling sound in the abdomen	−0.069	0.358	0.099	0.000	−0.087	−.180	**0.696**	0.286
Itching	0.101	−0.168	−0.040	0.059	0.244	0.053	0.187	**0.791**
**Variance explained (%)**	10.421	10.337	8.951	8.654	8.285	8.147	7.259	5.524

Symptoms with factor loading ≥0.4 are marked bold.

**Table 7:** Factor loadings for the items of the phlegm pattern questionnaire responded by the fourth-year students.

Item	Factor							
	1	2	3	4	5	6	7	8
Indigestion	**0.713**	−0.145	0.388	0.114	0.120	0.041	−0.110	0.048
Feeling of abdominal fullness	**0.705**	0.091	0.312	0.050	0.082	0.056	0.054	−0.050
Rumbling sound in the abdomen	**0.669**	0.359	−0.072	0.250	0.063	0.204	−0.067	−0.022
Sickness	**0.607**	−0.038	0.025	0.072	−0.001	−0.220	0.229	0.336
Startled by faint noise	0.047	**0.866**	−0.133	0.044	0.075	0.012	−0.153	0.060
Palpitation	0.077	**0.834**	−0.104	0.066	0.147	0.093	0.057	0.104
Tinnitus	−0.045	**0.613**	0.281	−0.048	0.342	0.066	0.324	−0.092
Itching	0.034	**0.437**	0.353	0.381	−0.164	0.315	0.022	0.158
Feeling heavy in the limbs	0.175	−0.047	**0.769**	0.210	−0.136	0.186	0.048	0.102
Fatigue	0.251	−0.132	**0.744**	0.164	0.191	0.039	−0.038	−0.022
Poor appetite	**0.509**	−0.042	**0.548**	0.087	0.152	0.057	0.256	0.078
Shortness of breath	0.039	0.210	**0.548**	0.118	−0.011	−0.202	0.257	**0.430**
Dark circles under the eyes	0.128	0.181	0.180	**0.762**	0.192	−0.044	−0.060	0.034
Yellowish face	0.146	−0.121	0.248	**0.726**	0.061	0.181	−0.031	0.198
Lumps	0.233	0.087	0.161	**0.525**	−0.171	0.184	0.347	−0.036
Unclearness in the head	0.299	0.047	0.089	−0.081	**0.747**	0.127	−0.062	−0.056
Dizziness	0.112	0.280	0.031	0.037	**0.730**	−0.009	0.222	0.019
Headache	−0.083	0.128	−0.020	0.315	**0.678**	0.155	−0.051	0.279
Mucousy stool	0.348	0.109	0.034	0.389	**−0.453**	0.284	0.093	−0.001
Flank pain	−0.047	0.238	0.046	0.019	0.033	**0.854**	0.079	0.087
Joint pain	0.132	−0.036	0.121	0.194	0.143	**0.762**	0.199	0.094
Feeling of foreign body in the throat	0.022	0.103	0.115	0.020	0.026	0.064	**0.828**	−0.010
Sputum	0.080	−0.238	−0.065	0.001	0.074	**0.430**	**0.670**	0.201
Cough	0.036	0.100	0.043	0.226	0.053	0.168	0.074	**0.804**
Feeling heavy in the chest	0.165	0.021	0.278	**−0.438**	0.136	0.192	−0.094	**0.563**
**Variance explained (%)**	10.257	10.146	9.905	9.009	8.597	8.165	6.890	6.042

Symptoms with factor loading ≥0.4 are marked bold.

**Table 8:** Factor loadings for the items of the phlegm pattern questionnaire responded by the fifth-year students.

Item	Factor								
	1	2	3	4	5	6	7	8	9
Indigestion	**0.832**	0.030	0.172	−0.015	−0.100	0.032	−0.085	−0.054	0.077
Sickness	**0.784**	0.014	−0.190	0.038	0.046	−0.036	0.054	0.024	0.053
Feeling of abdominal fullness	**0.743**	0.102	0.180	0.069	−0.051	−0.014	−0.032	0.079	0.033
Rumbling sound in the abdomen	**0.598**	0.172	−0.083	0.024	**0.400**	0.092	−0.054	−0.316	−0.223
Poor appetite	**0.571**	−0.165	**0.436**	0.082	0.048	0.022	−0.155	0.194	0.027
Palpitation	−0.073	**0.850**	−0.085	−0.031	−0.029	0.012	0.154	−0.235	0.148
Startled by faint noise	0.011	**0.759**	0.107	0.131	−0.053	0.001	0.095	0.027	0.153
Dark circles under the eyes	0.279	**0.624**	−0.054	0.223	0.010	−0.043	−0.173	0.298	−0.321
Dizziness	0.265	**0.500**	0.034	−0.030	−0.051	0.037	**0.411**	0.110	−0.395
Fatigue	0.025	0.124	**0.877**	−0.020	0.003	0.002	0.047	0.117	0.001
Feeling heavy in the limbs	0.145	−0.204	**0.754**	0.144	0.143	0.149	0.214	−0.203	0.054
Shortness of breath	0.309	0.396	**0.495**	−0.153	0.282	0.154	−0.235	−0.048	0.075
Lumps	0.076	−0.008	−0.056	**0.830**	0.176	0.075	0.027	0.154	−0.127
Yellowish face	−0.087	0.244	0.060	**0.729**	−0.106	0.068	0.054	0.056	0.354
Mucousy stool	0.380	−0.017	0.163	**0.497**	0.058	0.078	−0.015	−0.299	−0.135
Cough	−0.146	−0.059	0.191	−0.007	**0.853**	0.140	0.004	0.049	0.120
Sputum	0.132	−0.054	0.011	0.196	**0.754**	−0.248	−0.056	0.243	−0.165
Headache	−0.076	0.006	0.186	−0.051	−0.136	**0.790**	0.095	0.030	−0.168
Joint pain	0.100	−0.019	−0.052	0.198	0.073	**0.706**	0.032	0.074	0.155
Itching	0.065	0.107	0.357	0.366	0.293	**0.401**	−0.050	−0.267	0.077
Unclearness in the head	−0.165	0.013	0.178	0.136	−0.074	−0.057	**0.797**	0.079	0.125
Tinnitus	−0.024	0.329	−0.089	−0.129	0.055	0.341	**0.635**	−0.029	0.022
Flank pain	−0.048	0.033	0.160	0.102	0.020	0.386	−0.187	**0.653**	0.292
Feeling of foreign body in the throat	0.075	−0.058	−0.100	0.035	0.310	−0.059	0.275	**0.645**	0.005
Feeling heavy in the chest	0.162	0.147	0.053	0.021	−0.013	0.025	0.120	0.156	**0.844**
**Variance explained (%)**	12.367	9.733	8.846	7.417	7.358	6.854	6.218	5.962	5.783

Symptoms with factor loading ≥0.4 are marked bold.

**Table 9:** Factors of phlegm pattern questionnaire according to year of study and a previous study.

First year	Second year	Third year	Fourth year	Fifth year	Study by Park et al. [6]
Sputum, cough, feeling of foreign body in the throat, shortness of breath	Sputum, cough, feeling of foreign body in the throat	Sputum, cough, feeling of foreign body in the throat, shortness of breath, feeling heavy in the chest	Sputum, feeling of foreign body in the throat	Sputum, cough, rumbling sound in the abdomen	Sputum, cough, feeling of foreign body in the throat
Sickness, indigestion, poor appetite, lumps yellow face	Sickness, indigestion, Poor appetite, feeling of abdominal fullness, rumbling sound in the abdomen	Sickness, indigestion, poor appetite, feeling of abdominal fullness	Sickness, indigestion, poor appetite, feeling of abdominal fullness, rumbling sound in the abdomen	Sickness, indigestion, poor appetite, feeling of abdominal fullness, rumbling sound in the abdomen	Sickness, indigestion, poor appetite, feeling of abdominal fullness, rumbling sound in the abdomen
Rumbling sound in the abdomen, feeling of abdominal fullness		Mucousy stool, rumbling sound in the abdomen, feeling of abdominal fullness			
Headache, unclearness in the head, mucousy stool(−)	Headache, join pain, flank pain, unclearness in the head, lumps, feeling heavy in the chest feeling of abdominal fullness	Headache, unclearness in the head, lumps, dizziness, tinnitus	Headache, Unclearness in the head, dizziness, mucousy stool(−)	Headache, joint pain, itching	
Tinnitus, dizziness, flank pain, mucousy stool	Tinnitus, dizziness, sickness	Yellowish face, joint pain, flank pain	Joint pain, flank pain, sputum	Tinnitus, dizziness, unclearness in the head	
Fatigue, feeling heavy in the limbs, yellowish face	Fatigue, feeling heavy in the limbs, shortness of breath	Fatigue, feeling heavy in the limbs, flank pain	Fatigue, feeling heavy in the limbs, shortness of breath poor appetite	Fatigue, feeling heavy in the limbs, shortness of breath, poor appetite	Fatigue, feeling heavy in the limbs, headache, dizziness, unclearness in the head
Feeling heavy in the chest			Feeling heavy in the chest, cough, shortness of breath	Feeling heavy in the chest	
Palpitation, startled by faint noise	Palpitation, startled by faint noise, feeling heavy in the chest, poor appetite	Palpitation, startled by faint noise, tinnitus, sputum(−), shortness of breath	Palpitation, startled by faint noise, tinnitus, itching	Palpitation, startled by faint noise, dark circles under the eyes, dizziness	Palpitation, startled by faint noise, tinnitus, joint pain, flank pain, shortness of breath feeling, heavy in the chest
Itching, Joint pain, dark circles under the eyes	Itching, yellowish face, dark circles under the eyes, mucousy stool, rumbling sound in the abdomen	Itching	Dark circles under the eyes, yellowish face, lumps, feeling heavy in the chest(−)	Yellowish face, lumps, mucousy stool	Itching, lumps, mucousy stool
				Feeling of foreign body in the throat, flank pain	Dark circles under the eyes, yellow face

Only items with a factor loading ≥0.4 were extracted.

Factor order does not equal the order of variance explained.

(−) Factor loading is negative value.
