# Supplementary material for: Multivariate Analysis as a Method for Evaluating the Conceptual Perceptions of Korean Medicine Students regarding Phlegm Pattern
Source: Evid Based Complement Alternat Med. 2013 Aug 26;2013:761497. doi: 10.1155/2013/761497 (PMC3770024; doi:10.1155/2013/761497)
Supplement: Supplementary file 1 — This phlegm pattern questionnaire was developed in 2011 to evaluate a patient's phlegm pattern score and consists of 25 items. Researchers used Delphi method, factor analysis and ROC curves to develop and validate the questionnaire. [file 761497.f1.docx]

Supplementation

1. Table. 25-Item phlegm pattern questionnaire by Park’s previous study

| **Category** | **Condition** | **Symptom or sign** |
| --- | --- | --- |
| Neuropsychologic | I feel unclear in the head. | Unclearness in the head |
|  | I have a headache. | Headache |
|  | I feel dizzy. | Dizziness |
|  | I have ringing in the ears. | Tinnitus |
|  | I feel my heart palpitate. | Palpitation |
|  | I am startled by faint noise. | Startled by faint noise |
|  | I feel heavy in the chest. | Feeling heavy in the chest |
| Respiratory | I have a cough. | Cough |
|  | I have sputum in my throat. | Sputum |
|  | I feel a foreign body present in the throat, neither swallowed nor ejected. | Feeling of foreign body in the throat |
|  | I feel short of breath. | Shortness of breath |
| Fatigue-related | I feel fatigued. | Fatigue |
|  | I feel heavy or weak in the limbs. | Feeling heavy in the limbs |
|  | I have a poor appetite. | Poor appetite |
| Gastrointestinal | I feel sick to the stomach. | Sickness |
|  | I have indigestion. | Indigestion |
|  | I have a feeling of fullness in the stomach with just a little food. | Feeling of abdominal fullness |
|  | My stomach or intestine rumbles. | Rumbling sound in the abdomen |
|  | My stool is mucousy. | Mucousy stool |
| Dermatological | I have a lump somewhere on my body. | Lumps |
|  | My face is yellowish. | Yellowish face |
|  | I have dark circles under the eyes. | Dark circles under the eyes |
|  | I feel itchy. | Itching |
| Pain-related | I have pain in the joints. | Joint pain |
|  | I have flank pain. | Flank pain |
